# Isolation and characterisation of polychlorinated biphenyl (PCB) degrading fungi from a historically contaminated soil

**DOI:** 10.1186/1475-2859-8-5

**Published:** 2009-01-12

**Authors:** Valeria Tigini, Valeria Prigione, Sara Di Toro, Fabio Fava, Giovanna C Varese

**Affiliations:** 1 Department of Plant Biology, University of Turin, viale Mattioli 25, 10125 Turin, Italy; 2 DICASM, Faculty of Engineering, Alma Mater Studiorum-University of Bologna, viale Risorgimento 2, 40136 Bologna, Italy; 3 Marcopolo Engineering SpA, via XI Settembre 37, 12011 Borgo San Dalmazzo, Cuneo, Italy

## Abstract

**Background:**

Polychlorinated biphenyls (PCBs) are widespread toxic pollutants. Bioremediation might be an effective, cost competitive and environment-friendly solution for remediating environmental matrices contaminated by PCBs but it is still unsatisfactory, mostly for the limited biodegradation potential of bacteria involved in the processes. Very little is known about mitosporic fungi potential in PCB bioremediation and their occurrence in actual site historically contaminated soils. In the present study, we characterised the native mycoflora of an aged dump site soil contaminated by about 0.9 g kg^-1 ^of Aroclor 1260 PCBs and its changing after aerobic biotreatment with a commercial complex source of bacteria and fungi. Fungi isolated from the soil resulting from 120 days of treatment were screened for their ability to adsorb or metabolise 3 target PCBs.

**Results:**

The original contaminated soil contained low loads of few fungal species mostly belonging to the Scedosporium, Penicillium and Aspergillus genera. The fungal load and biodiversity generally decreased throughout the aerobic treatment. None of the 21 strains isolated from the treated soil were able to grow on biphenyl (200 mg L^-1^) or a mixture of 2-chlorobiphenyl, 4,4'-dichlorobiphenyl and 2,2',5,5'-tetrachlorobiphenyl (20 mg L^-1 ^each) as sole carbon sources. However, 16 of them grew in a mineral medium containing the same PCBs mixture and glucose (10 g L^-1^). Five of the 6 isolates, which displayed the faster and more extensive growth under the latter conditions, were found to degrade the 3 PCBs apparently without the involvement of ligninolytic enzymes; they were identified as Penicillium chrysogenum, Scedosporium apiospermum, Penicillium digitatum and Fusarium solani. They are the first PCB degrading strains of such species reported so far in the literature.

**Conclusion:**

The native mycoflora of the actual site aged heavily contaminated soil was mainly constituted by genera often reported as able to biodegrade organopollutants. It was generally remarkably reduced after the biotreatment, which however resulted in the selection of few mitosporic fungal species able to biodegrade PCBs. This is the first study in which an extensive characterisation of the cultivable indigenous mycoflora of an actual site aged PCB contaminated soil, as well as its changes upon soil bioremediation treatment, was conducted. Moreover, this is the first paper in which 5 strains ascribable to 4 mitosporic species able to biodegrade PCB are reported in the literature.

## Background

Polychlorinated biphenyls (PCBs) are highly toxic priority pollutants widespread in several former industrial sites and related terrestrial and aquatic habitats [1,2]. Such pollutants accumulate in the food chain where can exert toxic effects; thus, their removal from soils and sediments is a priority of great relevance in several industrialized Countries [2]. Bioremediation might be an effective, cost competitive and environment-friendly strategy for remediating soils contaminated by low concentrations of mixtures of low chlorinated PCBs [3-7]. Till now, most of the work has been focused on the characterisation of PCB degrading bacteria in soils and sediments [3-7] and addressed to stimulate soil bacterial activity through soil supplementation with oxygen, biphenyl, exogenous specialized bacteria and by treatment conditions able to provide a high degree of soil mixing and homogeneity [5-10].

On the contrary, little is known about the PCB biodegradation potential of fungi [11-14], despite of their well documented ability to biodegrade a large variety of aromatic priority pollutants [15] and their advantages over the bacterial systems in organopollutant removal from complex environmental matrices (i.e. production of robust nonspecific extracellular enzymes and free radical attack system, that can degrade poorly bioavailable or water insoluble complex mixtures of organic pollutants), that make them unique and of great potential in the bioremediation [16]. To the best of our knowledge no screenings and characterisation studies of fungi occurring in actual PCB contaminated soils have been conducted so far. In fact, the very few species known to degrade PCBs are principally ligninolytic fungi that have already been selected for the degradation of other aromatic pollutants: *Phanerochaete chrysosporium*, *Trametes versicolor*, *Lentinus edodes*, *Pleurotus ostreatus*, *Grifola frondosa *and *Coriolopsis polyzona *[12,17-19]. Whereas mitosporic fungi have been rarely considered for PCBs degradation despite of their biotransformation capabilities [20-22].

PCBs degradation by fungi is generally affected by several cultural conditions (i.e. medium composition, initial PCBs concentration, etc.) [2,13,15] and substantial amounts of PCBs can be abiotically removed through fungal mycelia sorbtion [23-25]. In addition, the fungal PCB degradation pathway has still not been completely elucidated. Only recently the mitosporic fungus *Paecilomyces lilacinus *and the yeast-like fungus *Trichosporon mucoides *were found to metabolize low chlorinated biphenyls through monohydroxylation of both the unsubstituted and the chlorinated aromatic ring of the molecules followed by the cleavage of hydroxylated ring system [21]. Literature also indicates the non-specific ligninolytic systems (e.g laccases and peroxidases) as the main putative enzymes involved in PCB degradation by fungi, and in particular by basidiomycetes [16,17]; however the involvement of other enzymes, such as mono- and dioxygenases, has been also hypothesized [11,26,27].

In a recent study, the effects of a not acclimated commercial consortium of exogenous microorganisms (Enzyveba) in the aerobic microbial decontamination of an highly and historically PCB contaminated soil was studied in laboratory-scale slurry- and solid-phase bioreactors [5]. A partial biodegradation of PCBs, along with a moderate decrease of the initial soil ecotoxicity, were observed in the not-inoculated reactors. Enzyveba markedly enhanced PCB-biodegradation rate and extent as well as the final soil detoxification, in particular under slurry-phase conditions [5]. Nothing is known about the nature and metabolic properties of indigenous mycoflora of the original soil and how it changed throughout the treatment, in the absence and the presence of Enzyveba and under the different conditions. The present study was undertaken to investigate all the potential role of the indigenous mycoflora in the observed decontamination of the soil. Moreover, fungi isolated at the end of the bioremediation trial were selected for biodegradation experiments towards 3 PCB congeners. PCBs decrease due to biosorption and/or biodegradation was evaluated and the production of laccase (LAC), Mn-dependent peroxidase (MnP), and Mn-independent peroxidase (MiP) was investigated.

## Results and Discussion

### Mycological characterisation of the actual site PCB historically contaminated soil

The site soil displayed a fungal load lower than 10000 colony forming units per g of soil dry weight (CFU g^-1^_dw_) and a total of only 16 fungal taxa (Table 1). Such a fungal load and biodiversity are about 10 times lower than those typically observed in unpolluted soils, where fungal concentrations of about 10^5 ^CFU g^-1 ^dry weight of soil due to 90–140 different species are detected [28,29]. This finding can be ascribed to the high soil content of PCBs and heavy metals [5], which might have caused a remarkable selection of indigenous soil mycoflora, as already observed by other authors in historically strongly contaminated soils [28,30-32]. However, the same finding is noteworthy since, according to the literature, micromycetes are very sensitive to high concentrations of PCBs and are often absent in matrices with over than 500 mg kg^-1 ^of such pollutants [33].

**Table 1:** Fungal entities isolated from the contaminated soil

**Fungal entities**	**MEA**	**PR478A**	**Mean of media**
*Acremonium strictum *W. Gams	42 (0.4%)	-	21 (0.2%)

*Aspergillus flavus *Link var. *flavus*	42 (0.4%)	-	21 (0.2%)

*Aspergillus fumigatus *Fresenius var. *fumigatus*	-	83 (0.9%)	42 (0.4%)

*Aspergillus versicolor *(Vuillemin) Tiraboschi	42 (0.4%)	54 (0.6%)	48 (0.5%)

DBB+ sterile ialine basidiomycetes	4 (0.0%)	-	2 (0.0%)

*Cladosporium herbarum *(Persoon) Link	-	42 (0.4%)	21 (0.2%)

Yeasts	4546 (45.8%)	1088 (11.4%)	2817 (28.9%)

*Paecilomyces marquandii *(Massee) S. Hughes	75 (0.8%)	-	38 (0.4%)

*Penicillium brevicompactum *Dierckx	13 (0.1%)	4 (0.0%)	9 (0.1%)

*Penicillium paneum *Frisvad (ined.)	88 (0.9%)	-	44 (0.5%)

*Penicillium phoeniceum *J.F.H. Beyma	42 (0.4%)	-	21 (0.2%)

*Penicillium waksmanii *K.M. Zalessky	42 (0.4%)	229 (2.4%)	135 (1.4%)

*Phomopsis *sp.	54 (0.5%)	-	27 (0.3%)

*Scedosporium apiospermum *(Saccardo) Saccardo	4892 (49.3%)	8046 (84.3%)	6469 (66.5%)

*Trichurus spiralis *Hasselbring	4 (0.0%)	-	2 (0.0%)

*Umbelopsis isabellina *(Oudemans) W. Gams	42 (0.4%)	-	21 (0.2%)

Total CFU g^-1^_dw_	9928	9547	9736

Fungal entities are expressed as number of colony forming units per g of soil dry weight (CFU g^-1^_dw_). In brackets is indicated the percentage of occurrence of the species with respect to the total CFU g^-1^_dw_. "DBB +" means positive to the reaction with Diazonium Blue B salts.

The soil mycoflora was dominated by mitosporic fungi (71%) and yeasts (29%) (Table 1). None of the isolates was able to produce decolourisation halos on PR478A. *Scedosporium apiospermum *(anamorphic state of *Pseudoallescheria boydii*) was dominant. This species is normally associated with heavily polluted environments [34] and often involved in the degradation of aromatic hydrocarbons [35-37]. Also the genera *Penicillium *and *Aspergillus *were relevant in terms of quality (i.e. number of species) and quantity (i.e. fungal load). Strains belonging to these two genera have been often isolated from polluted soils, and their remarkable degradative capability towards different organopollutants has been recently reported [22,38]. To the best of authors's knowledge, this is the first time that a mycological characterisation of an actual site soil heavily contaminated by PCBs and heavy metals is reported in the literature.

### Mycological characterisation of soil during the aerobic bioremediation treatment

The mycoflora occurring in the soil at the beginning and at the end of bioremediation (corresponding to the 3^rd ^and the 120^th ^day of treatment, respectively) under slurry and solid phase conditions, in the presence and the absence of Enzyveba (see Di Toro et al. [5] for more details on the process) was analysed using selective media. As Spearman test did not point out any significant difference among MEA and PR478A media, the results of the identification are presented as the mean of the data obtained from them (Table 2).

**Table 2:** Fungal entities isolated from bioreactors at the beginning and at the end of the biotreatment

	3 days				120 days			
Entities	A	B	C	*D*	A	B	C	*D*

*Acremonium curvulum *W. Gams					50 (0.4%)			

*Aspergillus foetidus *Thom & sRaper		50 (0.5%)						

*Aspergillus fumigatus *Fresenius var. *fumigatus*	100 (0.6%)	100 (1.0%)			50 (0.4%)			

*Aspergillus ochraceus *K. Wilhelm					100 (0.9%)			

*Aspergillus ustus *(Bainier) Thom & Church			211,5 (0.7%)					

Basidiomycete with clamp connections	450 (2.5%)	300 (3.0%)	5499 (17.2%)	4849 (18.8%)				

Basidiomycete with clamp connections and arthroconidia			211,5 (0.7%)					

Basidiomycete with clamp connections and chlamidospores				776 (3.0%)				

DBB+ avellaneous basidiomycete with arthroconidia			211,5 (0.7%)					

DBB+ dematiaceous basidiomycete	50 (0.3%)		423 (1.3%)	194 (0.8%)				

DBB+ ialinus basidiomycete with arthroconidia	250 (1.4%)	100 (1.0%)	634.5 (2.0%)	388 (1.5%)				

DBB+ ialinus basidiomycete with chlamidospores	100 (0.6%)	100 (1.0%)						

DBB+ ialinus basidiomycete morphotype 1		100 (1.0%)						

DBB+ ialinus basidiomycete morphotype 2	800 (4.5%)	750 (7.5%)	5710.5 (17.9%)	3685 (14.3%)				

DBB+ ialinus basidiomycete morphotype 3			423 (1.3%)					

*Beauveria bassiana *(Balsamo) Vuillemin							306 (3.6%)	

*Cladosporium chlorocephalum *(Fresenius) E.W. Mason & M.B. Ellis	500 (2.8%)	200 (2.0%)	846 (2.6%)	1357.5 (5.3%)				

*Cladosporium herbarum *(Persoon) Link	650 (3.7%)	50 (0.5%)	1480.5 (4.6%)	388 (1.5%)				

*Clitocybe *sp.			211.5 (0.7%)					

*Engyodontium album *(Limber) de Hoog			211.5 (0.7%)					

*Eurotium herbariorum *(F.H. Wiggers: Fries) Link	50 (0.3%)							

*Fusarium solani f. pisi *(Jones) Snyder & Hansen					50 (0.4%)			

*Geomyces pannorum *(Link) Sigler & J.W. Carmichael *var. pannorum*							306 (3.6%)	

*Microsporum gypseum *(E. Bodin) Guiart & Grigoraki					50 (0.4%)			

*Myrothecium roridum *Tode			211.5 (0.7%)					

*Penicillium aculeatum *Raper & Fennel	50 (0.3%)							

*Penicillium aurantiogriseum *Dierckx	50 (0.3%)		634.5 (2.0%)	1745.5 (6.8%)				

*Penicillium camemberti *Thom	100 (0.6%)							

*Penicillium chrysogenum *Thom	1350 (7.6%)	400 (4.0%)	4864.5 (15.2%)	582 (2.3%)	50 (0.4%)		6430 (75.0%)	

*Penicillium commune *Thom			211.5 (0.7%)					

*Penicillium crustosum *Thom				388 (1.5%)				

*Penicillium digitatum *(Persoon) Saccardo								1160 (12.5%)

*Penicillium glabrum *(Wehmer) Westling	1650 (9.3%)	250 (2.5%)	211.5 (0.7%)	1357.5 (5.3%)	50 (0.4%)			1160 (12.5%)

*Penicillium palitans *Westling	100 (0.6%)		2538 (7.9%)					

*Penicillium paneum *Frisvad (ined.)					50 (0.4%)			

*Penicillium paxilli *Bainier			211.5 (0.7%)					

*Penicillium polonicum *K.M. Zalessky	250 (1.4%)		211.5 (0.7%)					

*Penicillium purpurescens *Raper & Thom	550 (3.1%)						306 (3.6%)	

*Penicillium restrictum *J.C. Gilman & E.V. Abbott	150 (0.8%)							

*Penicillium solitum *Westling				2133.5 (8.3%)				2320 (25.0%)

*Penicillium spinulosum *Thom	900 (5.1%)							

*Penicillium waksmanii *K.M. Zalessky	50 (0.3%)	50 (0.5%)		1164 (4.5%)				

*Scedosporium apiospermum *(Saccardo) Saccardo	9400 (53.1%)	7500 (75.4%)	6557 (20.5%)	6400 (24.8%)	11250 (96.2%)	16150 (100%)	1225 (14.3%)	4641 (50.0%)

*Schizophyllum commune *Fries	50 (0.3%)			194 (0.8%)				

DBB-dematiaceous sterile mycelium	50 (0.3%)							

DBB-moniliaceous sterile mycelium	50 (0.3%)		211.5 (0.7%)	194 (0.8%)				

Total CFU g_(dw)_^-1 ^(mean ± st.dev)	17700 ± 6199 a*	9950 ± 3913 b*	31937 ± 17655 c*	25796 ± 14905 ac*	11700 ± 5371 a*	16150 ± 6277 b*	8573 ± 15568 c*	9281 ± 21817 c*
	
PCBs removal in mg kg^-1 ^(mean ± st.dev)					65 ± 4	28 ± 3	129 ± 8	74 ± 50

Fungal entities are expressed in number of colony forming units per g of soil dry weight (CFU g^-1^_dw_). In brackets is indicated the percentage of presence of the given species with respect to the total CFU g^-1^_dw_. A = Bioaugmented solid-phase bioreactors, B = Enzyveba-free solid-phase bioreactors, C = bioaugmented slurry-phase bioreactors, D = Enzyveba-free slurry-phase bioreactors. "DBB + or -" means positive or negative to the reaction with Diazonium Blue B salts. Different small letters indicate significant differences among fungal loads; * indicates significant differences among fungal loads after 3 days and after 120 days (Mann Whitney, P = 0.05). PCB depletion occurs in bioreactors is also reported.

After 3 days of aerobic treatment under slurry or solid phase conditions, the total fungal load of soil supplemented with nutrients and, when indicated, Enzyveba ranged from 9950 CFU g^-1^_dw _to 31937 CFU g^-1^_dw_. The highest fungal loads were observed in the slurry phase reactors (Table 2), probably for the higher degree of mixing achieved in these reactors than in the solid-phase ones [5]. The addition of Enzyveba generally resulted in a fungal load increase that was significant under solid state condition (Table 2). Nevertheless, the mycoflora observed in the bioaugmented reactors was not so abundant and diversified as expected on the basis of the fungal content of the inoculum applied [39]. This could be due to the toxic effects of soil pollutants towards very sensitive germinating propagules probably generated in the soil bioreactors during the first few days of treatment. A total of 48 entities were identified (Table 2). *S. apiospermum *was the dominant species in all the bioreactors, followed by basiodiomycetes which often caused decolourisation halos on Poly R 478. High loads of *Penicillium *spp. were also detected (Table 2).

At the end of the treatment, lower fungal loads and species numbers were generally observed (Table 2). No significant differences between inoculated and Enzyveba-free bioreactors were recorded, while higher fungal loads were generally observed under solid-phase conditions than the slurry phase ones, probably for long term unfavourable submerged mycelium growing conditions and the high toxic pollutant bioavailability guaranteed by the latter conditions with respect to the former ones.

All strains isolated from soil at the end of the treatment were ascribable to 14 species of mitosporic fungi, 7 of which came from the inoculum. *S. apiospermum *and *Penicillium *spp. were the dominant species (up to 100% in the soil resulting from solid-phase reactors and 75% in that resulting from slurry-phase ones). Noteworthy is the complete disappearance of basidiomycetes, since several fungi of this group are considered eligible candidates for PCBs degradation [12,17-19,25,40].

### Tolerance vs PCBs and growing capability of isolated fungi

None of the 21 strains isolated from the bioreactors at the end of the bioremediation experiment were able to grow on biphenyl (200 mg L^-1^), or on a mixture of 2-chlorobiphenyl, 4,4'-dichlorobiphenyl and 2,2',5,5'-tetrabiphenyl (each at 20 mg L^-1^) in the absence of any other carbon source (Table 3). Actually, fungi are rarely reported as PCB degraders in absence of alternative carbon source [12], although the growth capability on biphenyl has been considered an indicator of the ability to degrade PCBs [1,21]. Sixteen strains were able to growth in minimal medium with the 3 PCBs and glucose (10 g L^-1^), but less rapidly and extensively than the control with glucose only; this evidence analogously to others already reported in the literature [12,41] confirms PCBs ability to adversely affect fungal growth, thus permitting to hypothesize that tolerance towards PCBs is a reliable indication of fungal ability to cometabolize PCBs [2]. On the basis of these considerations, the 6 strains that displayed the most rapid and extensive growth on PCBs and glucose (*Aspergillus fumgatus*, *Penicillium chrysogenum*, *Penicillium digitatum*, *Fusarium solani *and two strains of *Scedosporium apiospermum*) were selected for further degradation experiments.

**Table 3:** Ability of the fungal isolates to grow on different carbon sources

**Fungal strains**	**Origin (bioreactors)**	**Growth on Glucose**	**Growth on Glucose + hexane**	**Growth on biphenyl**	**Growth on PCBs mix**	**Growth on PCBs mix + Glucose**
*Acremonium curvulum *MUT 598	A	+++	+++	-	-	-

*Aspergillus fumigatus *MUT 4026	A	++++	++++	-	-	+++

*Aspergillus ochraceus *MUT 675	A	+++	+++	-	-	+

*Beauveria bassiana *MUT 676	C	+++	+++	-	-	-

*Fusarium solani *MUT 4020	A	+++	+++	-	-	+++

*Geomyces pannorum *MUT 588	C	+++	+++	-	-	-

*Microsporum gypseum *MUT 631	A	+++	+++	-	-	-

*Penicillium digitatum *MUT 4079	D	++++	++++	-	-	+++

*Penicillium glabrum *MUT 594	D	++++	++++	-	-	++

*Penicillium chrysogenum *MUT 4021	C	++++	++++	-	-	++++

*Penicillium chrysogenum *MUT 4023	A	+++	+++	-	-	+

*Penicillium chrysogenum *MUT 4025	C	+++	+++	-	-	+

*Penicillium chrysogenum *MUT 610	C	++++	++++	-	-	++

*Penicillium paneum *MUT 649	A	+++	+++	-	-	++

*Penicillium purpurescens *MUT 583	C	+++	+++	-	-	++

*Penicillium solitum *MUT 610	D	++++	++++	-	-	-

*Scedosporium apiospermum *MUT 614	A	+++	+++	-	-	++

*Scedosporium apiospermum *MUT 631	B	++++	++++	-	-	+++

*Scedosporium apiospermum *MUT 637	A	+++	+++	-	-	++

*Scedosporium apiospermum *MUT 641	C	++++	++++	-	-	+++

*Scedosporium apiospermum *MUT 697	A	++++	++++	-	-	+

- no growth; + explorative growth consisting of few pellets; ++ evident mycelium in several pellets; +++ consistent mycelium in numerous pellets; ++++ consistent mycelium in homogeneous sporulating colony.

### PCB biotransformation potential of selected isolates

**Figure 1:**
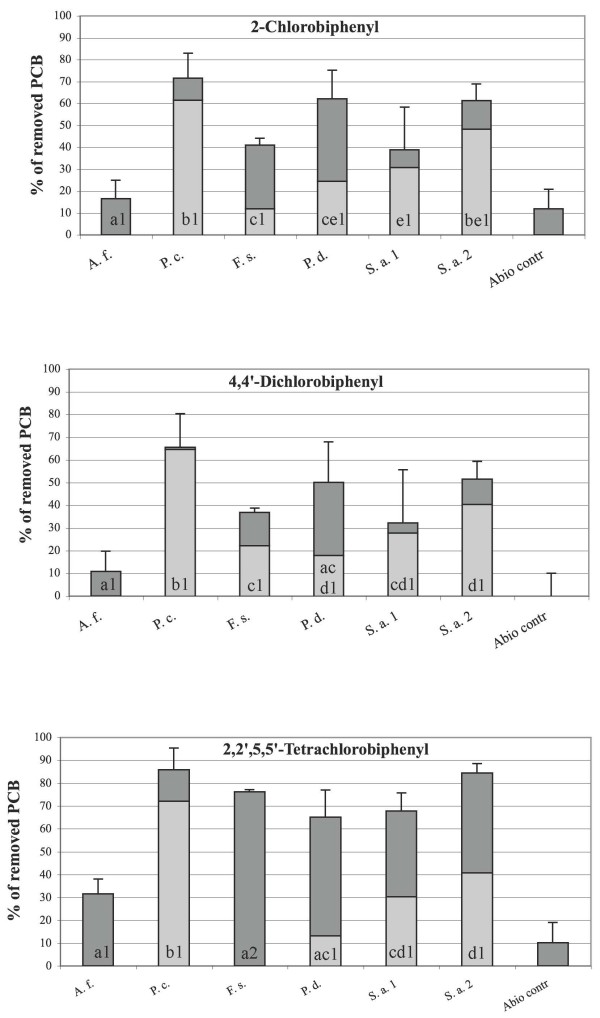
**Adsorption and biodegradation of target PCBs after 30 days of incubation**. PCB removal due to biodegradation (light grey); PCB removal due to biosorption (dark grey). A.f. = *Aspergillus fumigatus *MUT 4026; P.c. = *Penicillium chrysogenum *MUT 4021; F.s. = *Fusarium solani *MUT 4020; P.d. = *Penicillium digitatum *MUT 4079; S.a.1 = *Scedosporium apiospermum *MUT 641; S.a.2 = *Scedosporium apiospermum *MUT 631. Different small letters correspond to significant differences among PCBs biodegradation percentages achieved by different fungal strains towards the same congener. Different numbers correspond to significant differences among PCBs biodegradation percentages achieved by the same fungal strain towards different congeners.

The ability of the 6 selected fungi to remove 2-chlorobiphenyl, 4,4'-dichlorobiphenyl and 2,2',5,5'-tetrachlorobiphenyl in liquid mineral medium is shown in Figure 1. All the applied PCBs were remarkably removed (from 17% to 86%) by the fungi within 30 days of incubation. Considering that PCB recovery yields of the applied solvent extraction procedure was between 88 and 95% and that abiotic losses due to PCB volatilization associated to fungi cultivation were about 7%, it can be concluded that almost the whole observed PCB depletion yields were due to fungal biosorption and biodegradation processes [16,19,25,42]. To determine the role of the two processes to the final PCB removal observed, heat killed controls were developed and used to determine PCB sorption by each fungal strain. Nevertheless, inactivated fungal mycelia usually adsorb pollutants more effectively than living mycelia [43]. Indeed, biosorption of organopollutants and metal ions generally encompasses a number of metabolism-independent processes, still largely unknown [4,44], taking place essentially at the cell wall level [43,45] and differently in living and inactivated mycelia [46]. In the present study inactivated mycelia adsorbed the applied PCBs up to 3 times more efficiently than living mycelia (data not shown). Despite of this, the absorption data obtained were used to calculate the PCB biodegradation yields reported in Figure 1, even though this might have resulted in a remarkable underestimation of the biodegradation potential of the assayed cultures.

The PCB biosorption ability of 6 fungi was different and generally remarkable (Figure 1). *A. fumigatus *was the less efficient PCB removing strain and it seemed to exert its activity almost exclusively through biosorption. PCBs biosorption very often increased with the chlorination degree of biphenyl molecule; this finding, which is consistent with other evidences reported in the literature [25], indicates that the process is controlled by specific physical-chemical features of the molecule, such as steric conformation, charge distribution, polarity and water solubility, probably able to affect the overall pollutants-biomass binding sites interactions [44].

The PCBs biodegradation efficiency was very different among the 6 strains, indicating that different fungal species, but also different strains of the same species, metabolize congeners differently, as already pointed out by other authors [19,42,44]. *P. chrysogenum *almost always displayed the highest degradation potential against all the 3 PCB congeners (up to 72%). High PCB biodegradation yields (up to 48%) were also displayed by the two strains of *S. apiospermum*, whereas *P. digitatum *and *F. solani *showed moderate degradation capabilities (up to 24%) towards the 3 PCBs. Thus, 3 out of the 6 tested strains displayed a remarkable ability to biodegrade the 3 target PCBs, including 2,2',5,5'-tetrachlorobiphenyl, which is the highest chlorinated and most recalcitrant biphenyls among those applied. Notably, the same species have already been reported to degrade several organic xenobiotics [35,47-49], but they have never been quoted so far for their ability to degrade PCBs.

Unfortunately, all attempts to determine the main metabolites of PCB biodegradation in the cultures of the most active strains failed. In a preliminary experiment not described in the present paper, a prominent HPLC-detectable compound was found to temporarily accumulate in the culture of *P. chrysogenum*. Such a peak had retention times and diode array UV-absorption spectra very similar to those of pure chlorobenzoic acids; however, it did not co-eluted with any of chlorobenzoic acids we tested, i.e. 2-,3- and 4-monochlorobenzoic acids and 2,3-, 2,4-, 2,5-, 2,6-, 3,5-, and 3,4-dichlorobenzoic acids. The peak could not also be characterised as chlorocatechols or chloroacetophenons, which are other common aromatic intermediates of PCB aerobic bacterial degradation [3]. None significant net release of Cl^- ^was observed in the biologically active cultures of all PCB degrading strains. Thus, no information on the possible PCB biodegradation routes in the PCB biodegrading fungi described in this study are available.

### Enzymatic analysis

During PCB biodegradation experiments, only laccase activity was detected in the culture media of 6 assayed fungi. No MiP or MnP activities were detected in the same cultures, either in the presence or in the absence of PCBs, and this provides evidence that extracellular peroxidases were not involved in PCBs degradation. However, also the involvement of laccase in PCB biodegradation in the tested strains is not fully clear as it was produced extensively by *S. apiospermum *MUT 641 (up to 237 U L^-1^) and only in presence of PCBs but not by the parallel highly PCB biodegrading *S. apiospermum *strain MUT 631. On the other hand, biodegradation of aromatic compounds in these species have been so far ascribed to the production of dioxygenases [47]. *A. fumigatus, P. chrysogenum *and *P. digitatum *displayed low (up to 14 U L^-1^) and similar laccase activities irrespective of the presence of PCBs. *F. solani *also showed a moderate (up to 34 U L^-1^) and constant laccases activity throughout the whole experiment both in presence and the absence of PCBs.

Laccase activity has been already reported in other *A. fumigatus *[50] and *Penicillium *spp. [51] organopollutant biodegrading strains, but their direct involvement in pollutant degradation has been rarely reported [38]. Laccase production by *Fusarium *spp. seems to be constitutive [52], however organopollutants degradation by these species seems to be independent from laccase production [20].

Hence, for almost all fungi employed in this study, no clear correlation between PCBs degradation yields and tested ligninolytic enzymes production was observed and this suggests that also other enzymes, such as mono- or dioxygenase, can be involved in PCBs degradation by fungal isolates described in this work. Further studies will be addressed to investigate the involvement of laccases in PCB degradation by using purified enzymes.

## Conclusion

For the first time in the literature, the mycoflora of an actual site soil historically contaminated by high concentration of PCBs and heavy metals was characterised before, at the beginning and at the end of an aerobic biotreatment performed under laboratory conditions resembling those more commonly applied on the large scale on site remediation of PCB-contaminated soils. The original site soil showed a low concentration of few fungi. *Scedosporium apiospermum, Penicillium *spp. and *Aspergillus *spp. were the dominant species. The indigenous mycoflora was quantitatively and qualitatively further reduced during the biological treatment.

Nineteen of 21 mitosporic fungi isolated from the soil at the end of its biotreatment grew in the presence of a mixture of 2-chlrobiphenyls, 4,4'-dichlorobiphenyls and 2,2',5,5'-tetrachlorobiphenyl when also glucose was provided. Three of them, *P. chrysogenum *and the two *S. apiospermum *strains, never described as PCB degrader in the literature so far, extensively biodegrade the 3 target PCBs. This degradation, however, can not be clearly correlated with the tested ligninolytic enzymes. In addition, the data presented in this study provide evidence of the relevant role that indigenous fungal biomass might have on the final restoration of actual site historically PCB contaminated soils and of that fungal monitoring as well as fungal needs should be planned in designing an effective contaminated soil bioremediation strategy.

## Methods

### Soil characteristics

The actual site historically contaminated soil employed was strongly polluted by both medium-highly chlorinated biphenyls, largely attributable to Aroclor 1260, and heavy metals. Its main physical, chemical and microbiological characteristics as well the conditions under which it was subjected to biological treatment are reported by Di Toro and collaborators [5].

In brief, the soil was treated for 120 days under aerobic slurry and solid phase conditions in the absence and in the presence of Enzyveba [5], which is a complex consortium of bacteria and fungi patented and commercialized by Marcopolo Engineering Spa [39]. Soil samples collected at the beginning (after 3 days of treatment) and at the end of the experiment (120 days) were in this study subjected to mycological analysis and employed for fungi isolation and identification.

### Isolation of fungi from the soil before and after the bioremediation treatment

Samples of 10 g dry weight (dw) of site soil along with 10 g of dried soil resulting from bioreactors after 3 and 120 days of treatment were suspended in 90 ml Na_4_P_2_O_7_·10 H_2_O to disperse organic colloids; further dilutions were made in saline solution (9 g L^-1^NaCl). The final dilution (1:10000) was plated (1 ml per plate) on 20 replicates: 10 on generic MEA (20 g L^-1 ^malt extract, 20 g L^-1 ^glucose, 2 g L^-1 ^peptone, 18 g L^-1 ^agar) and 10 on the PR478A (20 g L^-1 ^malt extract, 20 g L^-1 ^glucose, 2 g L^-1 ^peptone, 18 g L^-1 ^agar, 0.2 g L^-1 ^Poly R478) containing the dye Poly R478, whose degradation by ligninolytic enzymes is considered predictive of the polycyclic aromatic hydrocarbons biodegradation capabilities [53]. Both media were supplemented with 15 mg L^-1 ^streptomycin sulphate and 50 mg L^-1 ^chloramphenicol to inhibit bacteria growth. Plates were incubated at 28°C and after about 3–4 days they were read to identify the colonies; serial readings were made to report decolourisation halos, to isolate and to track the progress of slowly growing fungi, for 4 weeks. The number of colony forming units per g of dry weight (CFU g^-1^_dw_) was calculated both for the total mycoflora and for each species or morphotype. Fungi were identified conventionally according to their macroscopic and microscopic features. After determination of their genera [54-56], they were transferred to the media recommended by the authors of selected genus monographs for species identification. Sterile mycelia were classified according to their hyphal pigments and their production of chlamydospores, sclerotia or vesicles. Sterile mycelia with clamp connections or positive to the reaction with Diazonium Blue B salts (DBB), according to Summerbell [57], were classified as basidiomycetes. When necessary, fungi identification was confirmed by the amplification and the direct sequencing of the internal transcribed spacers (ITS1 and ITS4) of the ribosomial DNA [58,59] and of the β-tubulins Bt2a and Bt2b [60].

The nonparametric Mann-Whitney test for independent groups was run to assess the significance (*p *≤ 0.05) of the differences among all thesis (media, incubation conditions, soil augmentation). The nonparametric Wilcoxon signed ranks test for dependent groups was run to assess the significance (*p *≤ 0.05) of the differences among sampling times (at the beginning and the end of the experiment). The nonparametric Spearman test was run to assess the significance (*p *≤ 0.05) of the quantitative and qualitative differences between the two media (MEA and PR478A).

### Tolerance and growing capability test

Fungal strains isolated from the bioreactors at the end of the bioremediation treatment were studied for their potential PCBs degradation capabilities. They were inoculated as a conidial suspension (10^5 ^conidia in 100 μl of sterile saline solution) in parallel 100 ml-flasks with Teflon liner-screw caps, containing 3 ml of a modified minimal medium MMM (1.7 g L^-1 ^ammonium nitrate, 2 g L^-1 ^KH_2_PO_4_, 0.5 g L^-1 ^MgSO_4_·7H_2_O, 0.157 g L^-1 ^CaCl_2_·4 H_2_O, 10 ml L^-1 ^micronutrient stock solution) with one of the following carbon sources: a) 10 g L^-1 ^glucose (Sigma, Milan, Italy), to check the vitality of the inocula; b) 0.2 g L^-1 ^biphenyl (Sigma, Milan, Italy), supplied by adding 18 μl of a 33.33 g L^-1 ^stock solution in hexane (Carlo Erba, Milan, Italy), to determine the ability of tested strains to metabolize biphenyl, which is the non-chlorinated analogue of PCBs generally used by PCB degrading microorganisms; c) a mixture of 2-Chlorobiphenyl, 4,4'-Dichlorobiphenyl and 2,2',5,5'-Tertachlorobiphenyl (Sigma, Milan, Italy), each at the final concentration of 20 mg L^-1^, supplied by adding 18 μl of a 10 g L^-1 ^stock solution in hexane (Carlo Erba, Milan, Italy), to determine the ability of tested strains to metabolize target PCBs; d) the PCBs mix mentioned above plus 10 g L^-1^glucose, to determine the ability of tested strains to co-metabolize PCBs; e) 10 g L^-1 ^glucose plus 18 μl hexane (the same solvent quantity utilised in the parallel cultures), to check the influence of the applied solvent on fungal growth; and f) MMN without carbon source, to check explorative fungal growth due to conidial energy reserves. Flasks were incubated at 25°C and 110 rpm for two weeks. Each trial was performed in triplicate.

### PCBs biodegradation in microcosms

From the previous growth test, 6 fungi were selected for further studies to determine their PCB biodegradation/biosorption potential. They were inoculated (10^5 ^conidia in 100 μl of distilled water) in 500 ml-flasks, containing 300 ml of EQ (20 g L^-1 ^glucose, 2 g L^-1 ^ammonium tartrate, 2 g L^-1 ^KH_2_PO_4_, 0.5 g L^-1 ^MgSO_4_·7H_2_O, 0.157 g L^-1^, Ca(NO_3_)_2_·4H_2_O, 10 ml L^-1 ^micronutrient stock solution), and incubated in the dark at 110 rpm and 25°C. After 7 days, biomasses were collected with a sieve (150 μm pore), homogenized and then rinsed and centrifuged in sterile distilled water five times, in order to remove residual C traces of the culture medium. Half of each biomass was inactivated in distilled water by autoclaving at 121°C for 30 min.

The static bioreactors were prepared by placing 0.5 g fresh weight (fw) of the wringed biomass of each fungus in 500 ml-flasks with Teflon liner-screw caps, containing 25 ml of MMM, supplemented with glucose (10 g L^-1^) and the mixture of 3 PCBs (final concentration: 20 mg L^-1 ^each). Heat-killed controls (composed by inactivated fungal biomass in the presence of PCBs) were obtained by preparing microcosms as described above, but inoculating them with 0.5 g fw of wringed inactivated biomass. Abiotic controls (PCBs without fungal biomass) and vitality controls (fungus with glucose and without PCBs) were also set up. Microcosms were incubated statically at 25°C in the dark for 30 days. The PCBs disappearance due to biodegradation was estimated at the end of the experiment subtracting the amount of PCBs not extracted from heat-killed control from the total PCBs removed by active mycelia. Each trial was performed in 6 replicates (3 for PCBs degradation and 3 for fungal growth evaluation and enzymatic analyses), with the exception of biotic and abiotic controls, performed in triplicate.

### Extractions and analytical procedures

At the end of the experiment PCBs were extracted from the entire microcosms (i.e., the mycelium and the liquid phase were solvent-extracted together) by using 21.5 ml of hexane and acetone mixture (9:1), vigorously agitated for 5 min and then sonicated for 10 min. The qualitative and quantitative analysis of PCBs occurring in the organic extracts was performed using a gas chromatograph (6890N), equipped with a HP-5 capillary column (30 mby 0.25 mm) and an electron capture detector (μECD) (Agilent Technologies, Inc. Life Sciences and Chemical Analysis Group, Santa Clara, CA, USA), according to the procedure described by Fava and collaborators [61]. The depletion of each added PCB was calculated from the average of 3 GC analyses of samples collected from the biological replicates at each given sampling time. Chlorobenzoic acids and related potential aromatic mononuclear intermediates of PCB biodegradation were batch extracted from acidified water phases by using diethyl-ether [62]. HPLC analysis of the obtained was performed with a Beckman HPLC system equipped with a Beckman ultrasphere 4.6 × 250 mm ODS column (5 μm particle diameter) and a 168 System Gold Diode Array detector operating at 235 and 254 nm (Beckman Instruments, Fullerton, CA, USA) as mentioned the same paper.

The concentration of Cl^- ^was monitored sampling 1.5 ml of culture medium from 3 parallel cultures after 1 day (T0), 15 days (T1) and 30 days (T2) of treatment and measuring on 0.2 μm pore-filtered samples by using a Dionex DX-120 IC system equipped with an IonPac AS14 4 × 250 mm column, a conductivity detector combined to a ASRS-Ultra conductivity suppressor system (Dionex Corporation, Sunnyvale, CA, USA). The eluent was a solution of 3.5 mM Na_2_CO_3 _and 1.0 mM NaHCO_3 _prepared in ultra-resi-analyzed water; the flow rate was 1.2 ml min^-1 ^and the injection volume was 20 μl. Chloride ion concentrations were determined by performing the average of the results obtained from the analyses of samples collected from the 3 parallel identical microcosms at each given sampling time.

### Enzymatic analysis

The activities of laccase (LAC), Mn-dependent peroxidase (MnP), and Mn-independent peroxidase (MiP) were monitored, sampling 1.5 ml of culture supernatants both from each flask with and without PCBs at T0, T1 and T2. Enzyme activities were measured spectrophotometrically (Amersham Biosciences Ultrospec 3300 Pro) using MBTH/DMAB as the chromogen (in the presence of Mn^2+ ^+ H_2_O_2_, in the presence of H_2_O_2 _without Mn^2+^, in the absence of both H_2_O_2 _and Mn^2+^, and in the presence of Na-citrate respectively). The MiP activity was calculated by subtracting reference values, and MnP activity by subtracting MiP values [63].

## Competing interests

The authors declare that they have no competing interests.

## Authors' contributions

VT carried out the experimental work described in the paper and drafted the manuscript. VP took part in the fungal identification and PCBs extraction, SDT performed the chemical analysis, FF and GCV coordinated the research as well as the manuscript preparation. All authors read and approved the final manuscript.
